# Supplementary material for: Geographical balancing of wind power decreases storage needs in a 100% renewable European power sector
Source: iScience. 2023 Jun 9;26(7):107074. doi: 10.1016/j.isci.2023.107074 (PMC10318522; doi:10.1016/j.isci.2023.107074)
Supplement: Document S1. Figures S1–S6 and Tables S1–S5 [file mmc1.pdf]

iScience, Volume *26*

Supplemental information

Geographical balancing of wind power decreases storage needs in a 100% renewable European power sector

Alexander Roth and Wolf-Peter Schill

SI. Supplemental Information

SI.1. Assumptions and data

SI.1.1. Time series

All time series concerning generation (capacity factors for solar PV, wind on- and off-shore, inflow series for hydropower plants) are taken from ENTSO-E's "Pan-European Climate Database (PECD)"[1]. The load data is taken from ENTSO-E's "Mid-term Adequacy Forecast (MAF) 2020"[2].

SI.1.2. Techno-economic parameters for technologies with endogenous capacities

Technology	Thermal efficiency [%]	Overnight investment costs [EUR/kW]	Technical Lifetime [years]
Bioenergy	0.487	1951	30
Run-of-river	0.9	600	25
PV	1	3000	50
Wind offshore	1	2,506	25
Wind onshore	1	1,182	25

Table SI.1: Technical and costs assumptions of installable generation technologies (related to STAR Methods)

Technology	Marginal costs of storing in [EUR/MW]	Marginal costs of storing out [EUR/MW]	Efficiency storing in [%]	Efficiency storing out [%]	Efficiency self-discharge [%]	Overnight investment costs in energy [EUR/kWh]	Overnight investment costs in capacity charge [EUR/kW]	Overnight investment costs in capacity discharge [EUR/kW]	Technical lifetime [years]
Lithium-Ion	0.5	0.5	92	92	100	200	150	150	13
Power-to-gas-to-power	0.5	0.5	50	50	100	1	3000	3000	20
Pumped-hydro	0.5	0.5	80	80	100	80	1100	1100	60
Reservoir	-	0.1	-	95	100	10	-	200	50

Table SI.2: Technical and cost assumptions of installable storage technologies (related to STAR Methods)

For the principal technical and cost parameters, we rely on previous research[3], and these are shown in Tables SI.1 and SI.2. For all technologies (generation and storage), we assume an interest rate for calculating investment annuities of 4%. The assumed power of installed bioenergy capacities is provided by ENTSO-E[4].

SI.1.3. Exogenous generation and storage capacities

Technology	Variable	AT	BE	CH	CZ	DE	DK	ES	FR	IT	NL	PL	PT
Bioenergy	Power [GW]	0.50	0.62	0	0.40	7.75	1.72	0.51	1.93	1.54	0.46	0.85	0.61
Run-of-River	Power [GW]	5.56	0.17	0.64	0.33	3.99	0.01	1.16	10.96	10.65	0.04	0.44	2.86
Pumped-hydro (closed)	Discharging power [GW]	0	1.31	3.99	0.69	6.06	0	3.33	1.96	4.01	0	1.32	0
	Charging power [GW]	0	1.15	3.94	0.65	6.07	0	3.14	1.95	4.07	0	1.49	0
	Energy [GWh]	0	5.30	670	3.70	355	0	95.40	10	22.40	0	6.34	0
Pumped-hydro (open)	Discharging power [GW]	3.46	0	0	0.47	1.64	0	2.68	1.85	3.57	0	0.18	2.95
	Charging power [GW]	2.56	0	0	0.44	1.36	0	2.42	1.85	2.34	0	0.17	2.70
	Energy [GWh]	1722	0	0	2	417	0	6185	90	382	0	2	1966
Reservoir	Discharging power [GW]	2.43	0	8.15	0.70	1.30	0	10.97	8.48	9.96	0	0.18	3.49
	Energy [GWh]	762	0	8155	3	258	0	11840	10000	5649	0	1	1187

Table SI.3: Assumptions on exogenous generation and storage capacities (related to STAR Methods)

SI.1.4. Interconnection capacities

link	Installed capacity [MW]
AT_CH	1700
AT_CZ	1100
AT_DE	7500
AT_IT	1470
BE_DE	1000
BE_FR	5050
BE_NL	4900
CH_DE	5300
CH_FR	4000
CH_IT	4850
CZ_DE	2300
CZ_PL	700
DE_DK	4000
DE_FR	4800
DE_NL	5000
DE_PL	3750
DK_PL	500
ES_FR	9000
ES_PT	4350
FR_IT	3255

Table SI.4: Installed Net Transfer Capacities (NTC) in model runs with interconnection (related to STAR Methods)

The assumed Net Transfer Capacities (NTC) provided in Table SI.4 are taken from from the TYNDP 2018 (Appendix IV - Cross-border capacities, NTC ST 2040)[4].

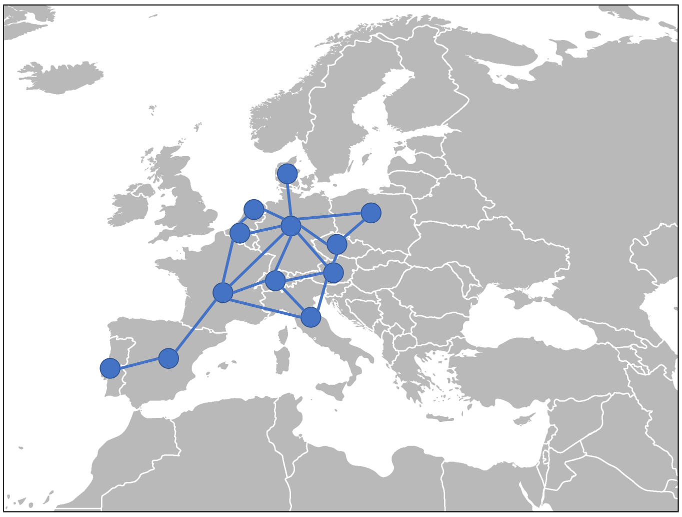

Figure SI.1: Geographic scope of the model and existing interconnections (related to STAR Methods)

Figure SI.1 depicts the countries that are part of the model and the respective interconnections between them.

SI.2. Model

Our analysis is model-based, using the open-source capacity expansion model DIETER. A short introduction is provided in section 4.3.3, more details are provided in previous publications[3,5].

For illustrative reasons, we provide below the formulation of two key equations of the model: the objective function and the energy balance. Before, we provide a non-exhaustive nomenclature of sets, variables, and parameters used in these equations. Variables are defined with uppercase letters and parameters with lowercase letters.

Sets. n is the set of countries, h the set hours, dis the set of dispatchable generators, nd the set of non-dispatchable generators, and sto the set of storage technologies.

Electricity generation and flows [MWh]. $G_{n,dis,h}$ is the generation of the dispatchable generation technology dis in country n in hour h. STO^{out} is the electricity generation (by discharge) of storage technologies, STO^{in} is the charging, and RSV^{out} is the electricity generation (by outflows) of reservoirs. $F_{l,h}$ is the electric energy sent over line l in hour h.

Installed generation capacities [MW]. N is the installed capacity of a generation technology. N^{p-out} is the installed discharging capacity of storage technologies, N^{p-in} is the installed charging capacity of storage technologies.

Energy installation variables [MWh]. N^e is the installed energy capacity of storage technologies.

Costs [Euro/MW(h)]. c^m are marginal costs of generation, c^i annualized investment costs of installation power and energy capacities (generation and storage), c^{fix} are the respective annual fixed costs.

Objective function. DIETER minimizes the total cost Z, consisting of variable generation costs (first term), investment costs of dispatchable and non-dispatchable generators (second term), as well as fixed and variable costs of storage (third term). The objective function of the model is given as:

$$
\begin{aligned}
Z = \sum_{n} \Bigg[& \sum_{h} \Big[\sum_{dis} c^{m}_{n,dis} G_{n,dis,h} + \sum_{sto} c^{m}_{n,sto} \left(STO^{out}_{n,sto,h} + STO^{in}_{n,sto,h} \right) \\
& + c^{m}_{n,rsv} RSV^{out}_{n,rsv,h} \Big] \\
& + \sum_{dis} \left[\left(c^{i}_{n,dis} + c^{fix}_{n,dis} \right) N_{n,dis} \right] + \sum_{nd} \left[\left(c^{i}_{n,nd} + c^{fix}_{n,nd} \right) N_{n,nd} \right] \\
& + \sum_{sto} \Big[\left(c^{i,p\text{-}out}_{n,sto} + c^{fix,p\text{-}out}_{n,sto} \right) N^{p\text{-}out}_{n,sto} + \left(c^{i,p-in}_{n,sto} + c^{fix,p-in}_{n,sto} \right) N^{p-in}_{n,sto} \\
& + \left(c^{i,e}_{n,sto} + c^{fix,e}_{n,sto} \right) N^{e}_{n,sto} \Big] \Bigg]
\end{aligned}
\tag{A.1}
$$

Those fixed variables (NTC capacities, installed capacities of hydro and bioenergy), and some nomenclature details, are omitted in the objective function for the reader's convenience. The full objective function is provided in the model code.

Energy balance. The wholesale energy balance reads as follows:

$$
\begin{aligned}
& d_{n,h} + \sum_{sto} STO^{in}_{n,sto,h} \\
& = \\
& \sum_{dis} G_{n,dis,h} + \sum_{nd} G_{n,nd,h} + \sum_{sto} STO^{out}_{n,sto,h} + \sum_{rsv} RSV^{out}_{n,rsv,h} \\
& + \sum_{l} i_{l,n} F_{l,h} \qquad \forall n, h
\end{aligned}
\tag{A.2}
$$

The left-hand side is total electricity demand in hour h at node n plus charging of storage technologies; the right-hand side is the total generation, including storage discharging, plus net imports: $F_{l,h}$ represents the directed flow on line l. If $F_{l,n} > 0$, electricity flows from the source to the sink of the line and reversed for $F_{l,n} < 0$. With the incidence parameter $i_{l,n} \in \{-1, 0, 1\}$, source, and sink are exogenously defined.

SI.3. Background on factorization

To identify the importance of different factors that reduce optimal storage need through interconnection, we (1) define several counterfactual scenarios and (2) then attribute the overall change to different factors using a "factorization" method[6,7].

To explain the principles of factorization, we borrow an example used in another paper[7]. Using a case study from the field of climate science, we aim to explain why

oceans around 3 million years ago were warmer than today. Assuming that two important factors are atmospheric CO_2 concentration and the extent and volume of large ice sheets, we apply a climate model and run several counterfactual scenarios. Both factors can have two kinds of states: CO_2 concentration can be low or high, and ice sheets can be small or large. Comparing different model outcomes, we can identify a "sole" CO_2 and ice sheet effect, but also an interaction effect between CO_2 concentration and ice sheet extension on ocean temperature.

Following the notation introduced in previous research[8], we describe the different scenarios in the following way: in f_0, ice sheets are small, and CO_2 is low. In the scenario f_1, the ice sheets are large, but CO_2 concentration is low. In scenario f_2, ice sheets are small, but CO_2 concentration is high. Finally, in scenario f_{12}, ice sheets are large, and CO_2 concentration is high.

The factorization method on which we rely on[6] defines the impact of the different factors in the following way:

$$\hat{f}_1 = f_1 - f_0, \tag{A.3}$$

$$\hat{f}_2 = f_2 - f_0. \tag{A.4}$$

$\hat{f}_1$ is the sole contribution of ice sheets, $\hat{f}_2$ of CO_2 concentration to the change in ocean temperature. However, with this factorization approach, the sum of the individual effects does not (in general) add up to the overall effect:

$$\hat{f}_1 + \hat{f}_2 \neq f_{12} - f_0 \tag{A.5}$$

Thus, an "interaction effect" $\hat{f}_{12}$ is introduced, which captures the joint effect of ice sheets size and CO_2 concentration on ocean temperature[6], such that $\hat{f}_1$, $\hat{f}_2$, and $\hat{f}_{12}$ add up to total the total effect $f_{12} - f_0$:

$$\begin{aligned} & f_{12} - f_0 = \hat{f}_1 + \hat{f}_2 + \hat{f}_{12} \\ \Leftrightarrow \quad & \hat{f}_{12} = f_{12} - f_0 - \hat{f}_1 - \hat{f}_2 \\ \Leftrightarrow \quad & \hat{f}_{12} = f_{12} - f_0 - (f_1 - f_0) - (f_2 - f_0) \\ \Leftrightarrow \quad & \hat{f}_{12} = f_{12} - f_1 - f_2 + f_0 \end{aligned} \tag{A.6}$$

If interested in the overall effect of CO_2 concentration and ice sheets on ocean temperatures and not in the interaction term, $\hat{f}_{12}$ has to be "distributed" to the other factors $\hat{f}_1$ and $\hat{f}_2$. This distribution can be done in different ways. One possibility is to share that interaction term equally between the two factors that are involved in that

interaction term. Following that logic, the total effect of the two factors becomes:

$$\hat{f}_1^{total} = f_1 - f_0 + \frac{1}{2}\hat{f}_{12} = \frac{1}{2}((f_1 - f_0) + (f_{12} - f2)) \tag{A.7}$$

$$\hat{f}_2^{total} = f_2 - f_0 + \frac{1}{2}\hat{f}_{12} = \frac{1}{2}((f_2 - f_0) + (f_{12} - f1)) \tag{A.8}$$

and capture the overall effect of ice sheets ($\hat{f}_1$) and CO_2 concentration ($\hat{f}_2$) on ocean temperatures. For a complete decomposition of factors, 2^n runs have to be conducted where n is the number of factors.

SI.4. Overview of scenario runs

Run	Identifier	(1) Interconnection	(2) Wind	(3) PV	(4) Load	(5) Hydro	(6) Bio
1	f_0	no	harmonized	harmonized	harmonized	harmonized	harmonized
2	f_1	yes	harmonized	harmonized	harmonized	harmonized	harmonized
3	f_2	no	not harmonized	harmonized	harmonized	harmonized	harmonized
4	f_3	no	harmonized	not harmonized	harmonized	harmonized	harmonized
5	f_4	no	harmonized	harmonized	not harmonized	harmonized	harmonized
6	f_5	no	harmonized	harmonized	harmonized	not harmonized	harmonized
7	f_6	no	harmonized	harmonized	harmonized	harmonized	not harmonized
8	f_{12}	yes	not harmonized	harmonized	harmonized	harmonized	harmonized
9	f_{13}	yes	harmonized	not harmonized	harmonized	harmonized	harmonized
...	...	...	...	...	...	...	...
63	f_{23456}	no	not harmonized	not harmonized	not harmonized	not harmonized	not harmonized
64	f_{123456}	yes	not harmonized	not harmonized	not harmonized	not harmonized	not harmonized

Table SI.5: Overview of scenario runs (related to STAR Methods)

Table SI.5 provides an intuition of which scenario runs are performed and how they are defined. For every weather year, 64 runs are needed for a complete factorization.

SI.5. Further results

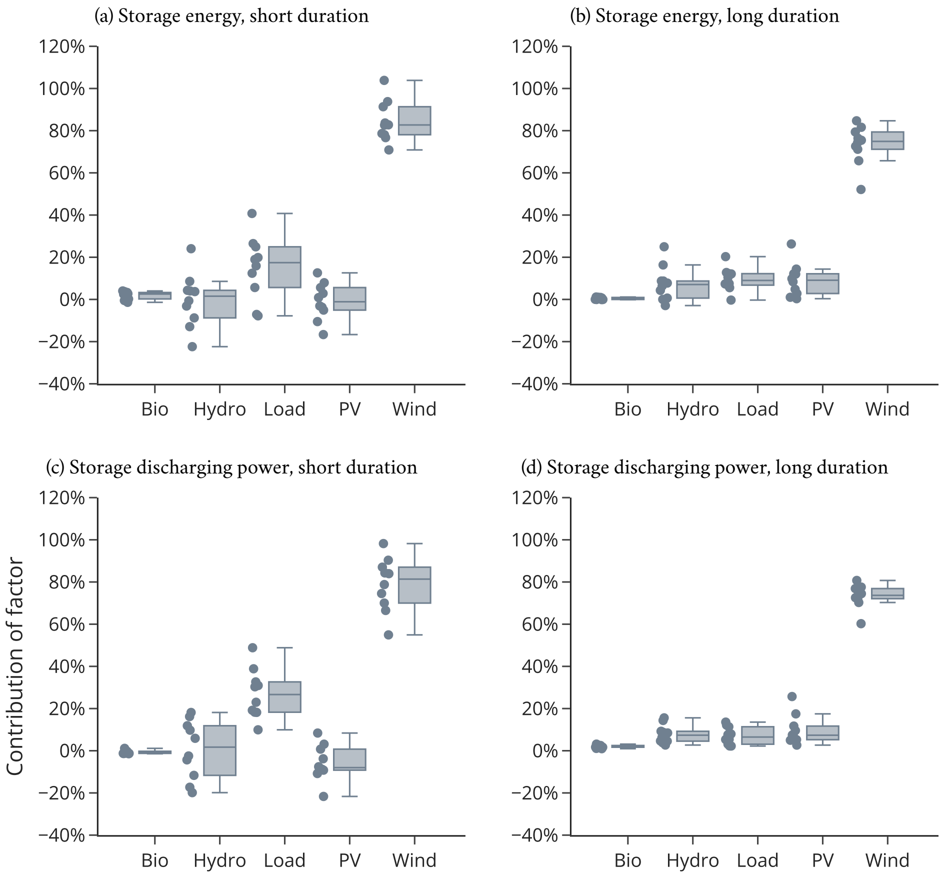

Notes: Every dot is the scenario result based on one weather year. The middle bar shows the median value. The box shows the interquartile range (IQR), which are all values between the 1st and 3rd quartile. The whiskers show the range of values beyond the IQR, with a maximum of 1,5 x IQR below the 1st quartile and above the 3rd quartile.

Figure SI.2: Relative contribution of different factors to the change in storage energy and discharging power capacity related to interconnection (related to Figure 2)

Heterogeneity in wind power explains between 55% and 104% of short-duration storage energy and discharging power capacity reduction and 52% to 85% of long-duration storage capacity reductions, respectively. At the other end of the spectrum, country-specific differences in installed bioenergy hardly have an effect. Differences in hydropower, load time series, and PV profiles have varying contributions, especially for short-duration storage. The effect of hydropower ranges between -22% and +24% for storage energy and -20% and +18% for storage discharging power (Figure SI.2).

We find similar outcomes for PV. The effect of different PV capacity factors through

interconnection on aggregate optimal short-duration storage energy or discharging capacity varies between -17% and +13%, or -22% and 8%, respectively. This contrasts with the results for wind power, which always decreases storage needs.

Negative percentage values indicate that the current heterogeneous mix of hydro capacities (run-of-river, reservoirs, and pumped hydro) may even increase optimal storage needs compared to a setting with equal relative shares, thus harmonized installations. Exploring this combined technology effects in detail merits further investigation.

Overall, the influence of different weather years on the composition of the factors is more pronounced for short-duration than for long-duration storage.

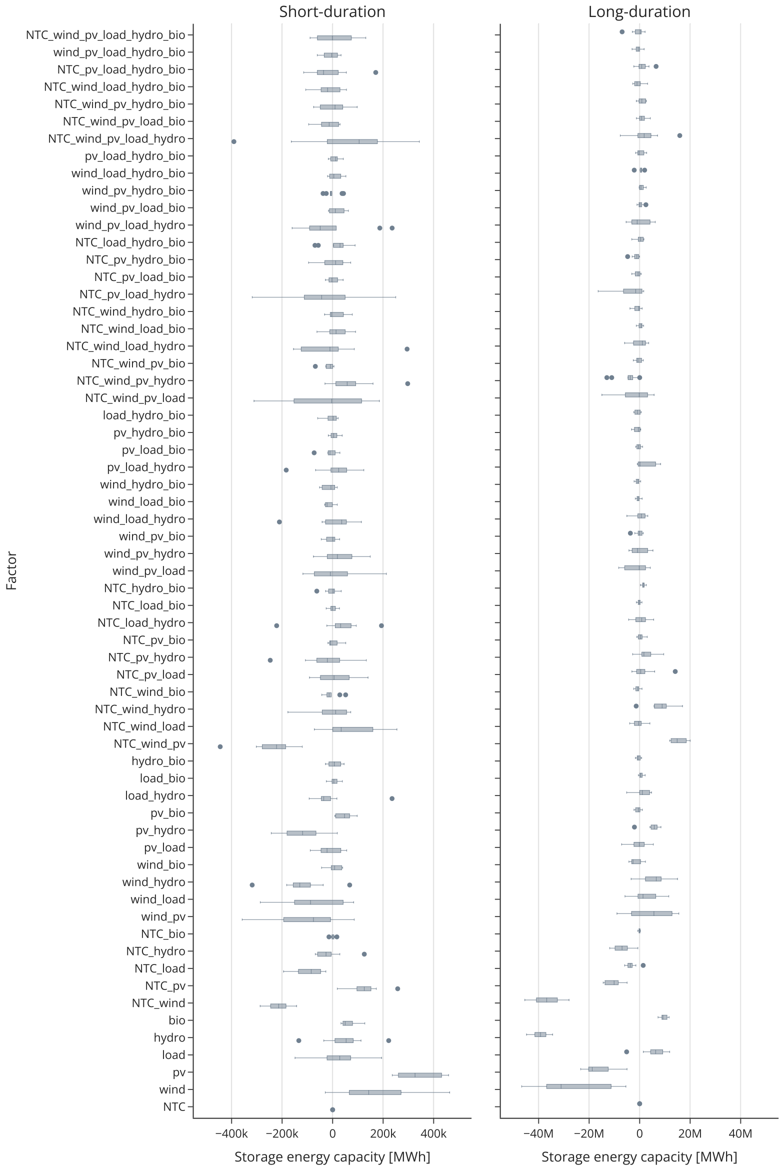

Figure SI.3: Impact of all factors on storage energy capacity in all years (related to Figure 2)

Notes: Differentiated by short- and long-duration, the strength of each individual factor is depicted, covering all 10 weather years. If below zero, a factor negatively impacts aggregate optimal storage energy capacity. If above zero, a factor increases aggregate optimal storage energy capacity.

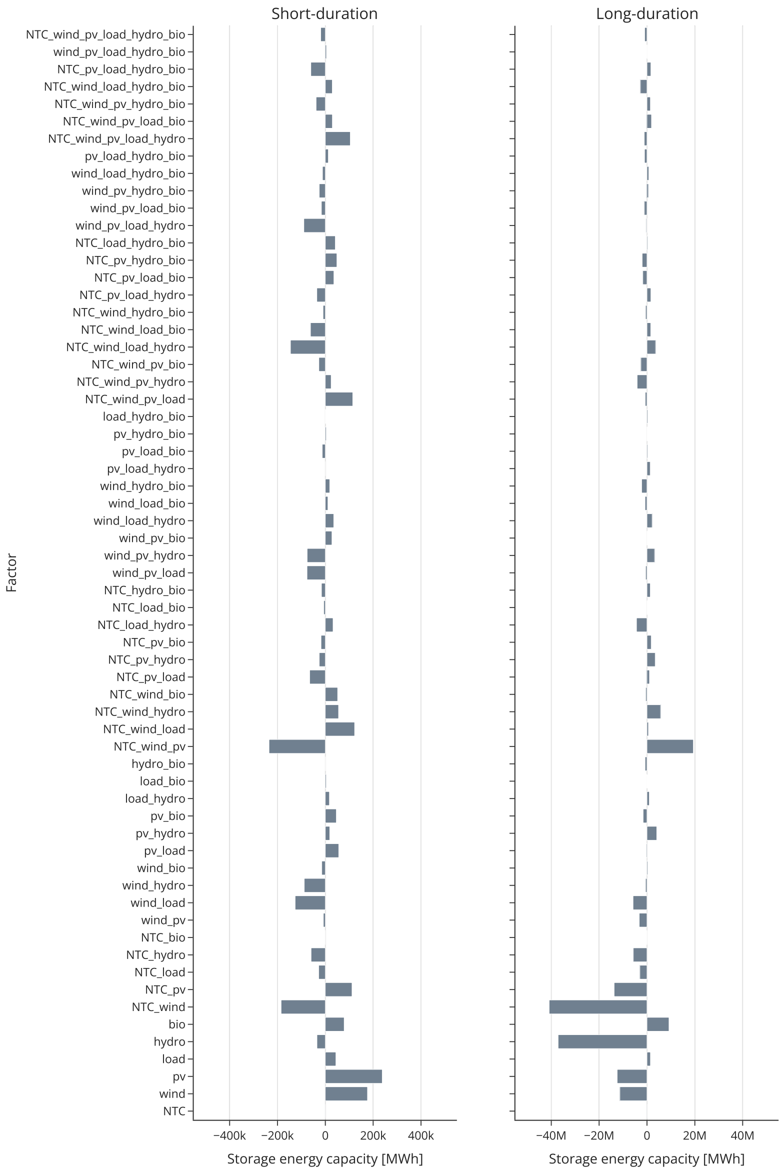

Figure SI.4: Impact of all factors on storage energy capacity in 2016 (related to Figure 2)

Notes: Differentiated by short- and long-duration, the strength of each individual factor is depicted for the weather year 2016. If below zero, a factor negatively impacts aggregate optimal storage energy capacity. If above zero, a factor increases aggregate optimal storage energy capacity.

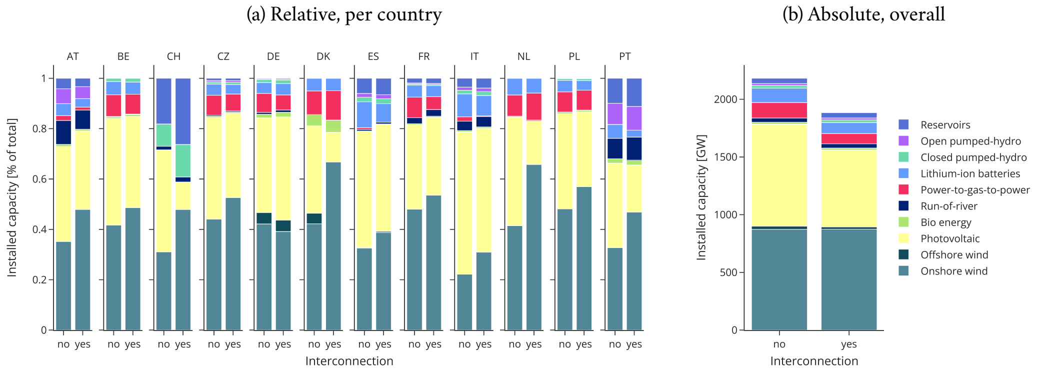

Figure SI.5: Installed power plant and storage discharging capacities for scenarios with or without interconnection (related to Figure 2)

Figure SI.5 shows optimal generation and storage capacities for scenarios with and without interconnection. While solar PV and onshore wind power dominate the capacity mix in all countries, the share of onshore wind power increases in scenarios with interconnection compared to the setting with isolated power systems in all countries (left panel). This further corroborates our conclusion that geographical balancing particularly helps to smooth wind power variability across countries. The Figure also shows that the overall generation capacity decreases in a setting with interconnection (right panel). This is largely driven by a lower need for solar PV generation capacity, enabled by lower curtailment and better (cross-border) use of installed wind power capacities.

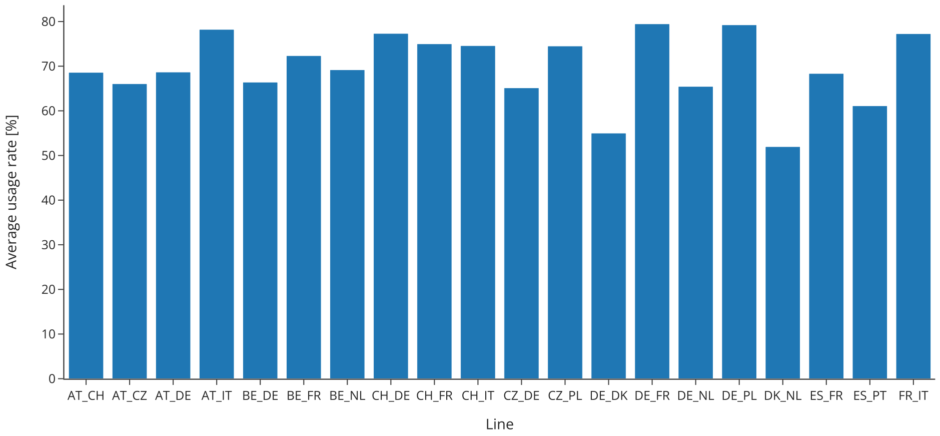

Notes: Data of the weather year 2016 shown.

Figure SI.6: Average hourly usage rates of interconnections (related to STAR Methods)

Average utilization rates of the modeled interconnections are both relatively high and homogeneous, with values between around 50% and 80% (Figure SI.6). Such high usage rates imply that the NTC expansion assumed by ENTSO-E[4] for 2040 may not be sufficient for the fully renewable central European power sector modeled here. The connections between Germany and its neighbors France, Poland, and Switzerland, as well as the lines between Austria and Italy and between France and Italy, are most heavily used. This indicates that further extensions of these connections would be particularly desirable. In contrast, interconnections between Denmark and the Netherlands as well as Denmark and Germany are relatively under-utilized.

References

[1] De Felice, M. (2020). ENTSO-E PECD (European Climate Database) from MAF 2019 in CSV and Feather formats. 10.5281/zenodo.3702418

[2] ENTSO-E. (2020). Mid-term Adequacy Forecast (MAF) 2020. https://eepublicdownloads.entsoe.eu/clean-documents/sdc-documents/MAF/2020/MAF_2020_Executive_Summary.pdf

[3] Gaete-Morales, C., Kittel, M., Roth, A., & Schill, W.-P. (2021). DIETERpy: A Python framework for the Dispatch and Investment Evaluation Tool with Endogenous Renewables. SoftwareX, 15, 100784. 10.1016/j.softx.2021.100784

[4] ENTSO-E. (2018). TYNDP 2018 - Appendix. https://eepublicdownloads.blob.core.windows.net/public-cdn-container/clean-documents/tyndp-documents/TYNDP2018/consultation/Main%20Report/TYNDP18%20Exec%20Report%20appendix.pdf

[5] Zerrahn, A., & Schill, W.-P. (2017). Long-run power storage requirements for high shares of renewables: Review and a new model. Renewable and Sustainable Energy Reviews, 79, 1518–1534. 10.1016/j.rser.2016.11.098

[6] Stein, U., & Alpert, P. (1993). Factor Separation in Numerical Simulations. Journal of the Atmospheric Sciences, 50(14), 2107–2115. 10.1175/1520-0469(1993)050<2107:FSINS>2.0.CO;2

[7] Lunt, D. J., Chandan, D., Haywood, A. M., Lunt, G. M., Rougier, J. C., Salzmann, U., Schmidt, G. A., & Valdes, P. J. (2021). Multi-variate factorisation of numerical simulations. Geoscientific Model Development, 14(7), 4307–4317. 10.5194/gmd-14-4307-2021

[8] Schär, C., & Kröner, N. (2017). Sequential Factor Separation for the Analysis of Numerical Model Simulations. Journal of the Atmospheric Sciences, 74(5), 1471–1484. 10.1175/JAS-D-16-0284.1
